# Deviations from additivity in *APOE4*-mediated late-onset Alzheimer’s disease risk across races and ethnicities

**DOI:** 10.1007/s00439-025-02810-5

**Published:** 2026-01-22

**Authors:** Razaq O. Durodoye, Timothy H. Ciesielski, Penelope Benchek, Jacquelaine Bartlett, Xiaofeng Zhu, Shiying Liu, Adam Naj, Brian Kunkle, Gerard D. Schellenberg, Richard Mayeux, Lindsay Farrer, Li-San Wang, Margaret A. Pericak-Vance, Farid Rajabli, Giuseppe Tosto, Jonathan L. Haines, William S. Bush, Scott M. Williams

**Affiliations:** 1 Department of Population and Quantitative Health Sciences, Case Western Reserve University School of Medicine, Cleveland, USA; 2 Department of Pathology and Laboratory Medicine, University of Pennsylvania Perelman School of Medicine, Philadelphia, USA; 3 The Dr. John T. Macdonald Foundation Department of Human Genetics, University of Miami Miller School of Medicine, Miami, USA; 4 Department of Neurology, Columbia University Irving Medical Center, New York, USA; 5 Department of Biostatistics, Boston University School of Public Health, Boston, USA

## Abstract

*APOE*’s ε4 haplotype (*APOE4*) is late onset Alzheimer’s disease’s (LOAD) strongest genetic risk factor. Therefore, accurately modeling *APOE4*’s effect is critical to understanding LOAD. This is especially important as *APOE4* odds ratios (OR) vary across racial and ethnic (R/E) groups. We analyzed the *APOE4*-LOAD association in 3,196 East Asian, 31,105 non-Hispanic White (White), 1,646 Hispanic and Latino (Hispanic), and 6,068 non-Hispanic Black (Black) participants using three genetic models: additive, genotypic, and a reparametrized model accounting for deviations from additivity (DA). Each model adjusted for age, sex, and genetic ancestry. We first calculated additive *APOE4* ORs in each R/E group, finding East Asian participants had the largest *APOE4* ORs (OR_*APOE4*_: 5.2, 95%CI: 4.4–6.0) and Black participants among the smallest (OR_*APOE4*_: 2.8, 95%CI: 2.6–3.1). Next, we generated *APOE4* ORs for heterozygote and homozygote ε4 carriers. These genotypic ORs were statistically the same as the additive *APOE4* estimates for all models except homozygote East Asian participants. The measured homozygote East Asian estimate (OR_*APOE4*_: 41.5, 95%CI: 22.1–88.8) was 57% higher than its predicted counterpart based on additivity, indicating significant non-additive contributions. Finally, we used a reparametrized model that adjusted for DA. Although equivalent to the genotypic model, this adjustment provided insight into DA while significantly improving model performance among Black participants. This report demonstrates that *APOE4* estimates differ based on the genetic modeling strategy, introduces explicit DA adjustments in the *APOE4*-LOAD association, and cautions against blindly assuming additivity across R/E groups.

**Supplementary Information:**

The online version contains supplementary material available at 10.1007/s00439-025-02810-5.

## Introduction

Apolipoprotein E (*APOE*) represents the strongest genetic risk factor for late-onset Alzheimer’s disease (LOAD), the most common form of dementia (2024; Mayeux and Stern 2012; Morris et al. 2019; Rajan et al. 2017; Rizzi et al. 2014; Tiwari et al. 2019). Extensive recruitment and research efforts involving non-Hispanic White and/or European-descent individuals (hereafter referred to as White) have estimated *APOE*’s attributable risk as high as twenty percent in some Northern European populations (Qiu et al. 2004). As this gene can account for a large proportion of genetic LOAD risk, accurately modeling *APOE*’s risk-increasing ε4 allele (*APOE4*) is critical in understanding risk (Bellenguez et al. 2022; Belloy et al. 2023; Breitner et al. 1999; Farrer et al. 1997; Kamboh et al. 2012; Qiu et al. 2004).

The characterization of *APOE4* in non-European descent populations has also documented this variant’s contribution to LOAD (Barnes et al. 2005; Belloy et al. 2023; Farrer et al. 1997; Morris et al. 2019; Rajabli et al. 2025; Rajabli et al. 2018; Rajan et al. 2017; Weiss et al. 2021). Prior reports showed that East Asian and East Asian American (hereafter referred to as East Asian) individuals have the largest *APOE4* odds ratios (OR), followed by White, non-Hispanic Black and/or African American (hereafter referred to as Black), and Hispanic and genetically admixed Latino (hereafter referred to as Hispanic) individuals. While the population with the lowest *APOE4* OR varies between Hispanic and Black groups by study, *APOE4* ORs in these two populations are consistently smaller compared to White and East Asian *APOE4* estimates.(Belloy et al. 2023; Farrer et al. 1997) In addition to these racial differences in the estimated effect of APOE4, there are also racial differences in the frequency of APOE4 (Asian: 9%, Hispanic: 12%, White: 14%, African: 19% (Jia et al. 2020)), and LOAD risk (Cumulative 25-year risk of dementia at age 65: 38% African-Americans, 32% Latino, 30% White, 28% Asian-Americans (Mayeda et al. 2016). African individuals appear to have the highest risk of LOAD and the highest frequency of APOE4, but the lowest APOE4 effect size. Asian individuals appear to have the lowest risk of LOAD and the lowest frequency of APOE4, but the highest APOE4 effect size. Finally we know that, local ancestry surrounding the *APOE* gene region is contributing to ε4 effect size variation across racial and ethnic (R/E) populations (Barnes et al. 2005; Belloy et al. 2023; Farrer et al. 1997; Rajabli et al. 2022, 2023; Rajabli et al. 2018; Rajan et al. 2017).

The effects of *APOE4* alleles on LOAD risk are often assumed to act additively, with each ε4 allele increasing risk proportional to its copy number, despite reports supporting deviations from additivity (DA) (Corder, et al. 1993; Fortea, et al. 2024; Ohta, et al. 2024; Tsouris, et al. 2024). Refining genetic modeling strategies to account for DA in the *APOE4*-LOAD association remains important as estimates including this adjustment may more accurately represent *APOE4*’s effect (Fortea, et al. 2024; Ohta, et al. 2024; Tsouris, et al. 2024). We explicitly evaluated DA by reparametrizing existing genetic models, assessed the impact of this adjustment on model performance, and how considering DA in our model affects the relative population specific conferred risk in multiple R/E groups.

## Methods

To confirm population specific *APOE4* risk and examine DA effects, we used data from the Alzheimer’s Disease Genetic Consortium’s (ADGC) (Kuzma, et al. 2016). The ADGC provided genetic and covariate information on 65,952 unique individuals from 43 cohorts. We addressed population variability in the *APOE4*-LOAD association by stratifying individuals into non-overlapping East Asian, White, Black, and Hispanic groups using R/E descriptors documented by the ADGC (Supplemental Table 1). After implementing quality control measures and removing third degree or closer relatives (kinship cutoff Φ_ij_ ≥ 0.0884), 42,015 participants remained in the final analytic dataset: 3,196 East Asian, 31,105 White, 6,068 Black, and 1,646 Hispanic individuals (Table 1, Supplemental Fig. 1). ADGC data is available upon request from the Consortium (https://www.adgenetics.org/content/feedback-and-queries). The study was approved by the Case Western Reserve University IRB.

**Table 1:** Study population demographics

		East Asian (n = 3,196)		White (n = 31,105)		Hispanic (n = 1,646)		Black (n = 6,068)	
		Case (n = 1,419)	Control (n = 1,777)	Case (n = 14,868)	Control (n = 16,237)	Case (n = 665)	Control (n = 981)	Case (n = 1,797)	Control (n = 4,271)
Age (Median, IQR)		75.0 (71.0, 78.0)	76.0 (72.0, 80.0)	78.0 (72.0, 83.0)	76.0 (70.0, 82.0)	79.0 (73.0, 85.0)	71.0 (66.0, 77.0)	80.0 (75.0, 85.0)	77.0 (70.0, 82.3)
Sex %Female (n)		71.0% (1,008)	53.0% (942)	57.3% (8,512)	59.7% (9,690)	64.2% (427)	71.9% (705)	69.3% (1,245)	72.1% (3,080)
*APOE4*	XX	44.0% (624)	82.3% (1,463)	39.6% (5,883)	74.0% (12,013)	57.1% (380)	79.1% (776)	39.8% (715)	64.2% (2,743)
	X4	45.5% (645)	17.2% (305)	47.1% (7,007)	24.0% (3,901)	34.0% (226)	19.6% (192)	47.9% (860)	32.9% (1,406)
	44	10.6% (150)	0.5% (9)	13.3% (1,978)	2.0% (323)	8.9% (59)	1.3% (13)	12.4% (222)	2.9% (122)

R/E population descriptors from ADGC

*XX APOE4* non-carriers, *X4* participants carrying one copy of *APOE4*, *44* participants carrying two copies of *APOE4*

Participants diagnosed with mild cognitive impairment were excluded from this study so that all comparisons are binary: cognitively unaffected participants (controls) or individuals affected by LOAD (cases). All participants were evaluated to determine case or control status based on the National Institute of Neurological and Communicative Disorders and Stroke-Alzheimer’s Disease and Related Disorders Association criteria (Albert et al. 2011; McKhann et al. 1984). Each remaining participant was 60 years of age or older, had a documented age of onset (case) or age at last assessment (control), and demographic information. To ensure high quality data for *APOE* genotypes*,* rs429358 and rs7412, the sites that define *APOE4*, were measured with the Taqman Assay as previously described (Rajabli et al. 2025). Principal components (PCs) were calculated using variants initially filtered at the cohort level to only include variants with minor allele frequency (MAF) > 1% in all R/E populations. Imputation was done using the Trans-Omics for Precision Medicine (TOPMed) Imputation Server (TIS) as previously published (Rajabli et al. 2025); in brief, we required imputation R^2^ > 0.8 with MAF between 1 and 5% and imputation R^2^ > 0.4 for variants with MAF > 5%. At the population level, variants were further filtered for linkage disequilibrium and only variants with pairwise R^2^ < 0.2 within 50 kb blocks were included, using PLINK 1.9.(Purcell et al. 2007) The final set of variants selected for PC calculation were based on the overlapping, filtered SNP set across all cohorts and all populations. In total, 142,005 genetic variants were retained for the calculation of PCs. We adjusted for global genetic ancestry within each R/E group by incorporating PCs into the regression models and determined that including the first two PCs corrected for within-group genetic admixture (Supplemental Fig. 2). We also performed sensitivity analyses to assess how varying the number of PCs affected *APOE4* ORs (Supplemental Fig. 3). PCs were calculated separately for each R/E group. ORs were compared using a Wald-test, with significance assessed at α = 0.05. Three main models were evaluated (Supplemental Eqs. 1, 2, and 3). A sensitivity analyses was conducted to explore the impact of potential differences by study site, by adding a random intercept for study site to Supplemental Eq. 3. To explore the role of sex we conducted two additional sensitivity analyses. In the first approach we added a DA-sex interaction term Supplemental Eq. 3 to determine if the DA term differed by sex. In the second approach we reran Supplemental Eq. 3 within the 8 strata defined jointly by sex and R/E. All significance tests were two sided Wald tests conducted at α = 0.05, and no corrections were made for multiple testing. Statistical analyses were conducted using R, version 4.4.1 (R Project for Statistical Computing)[Team 2024].

## Results and discussion

### Additive model

**Fig. 1:**
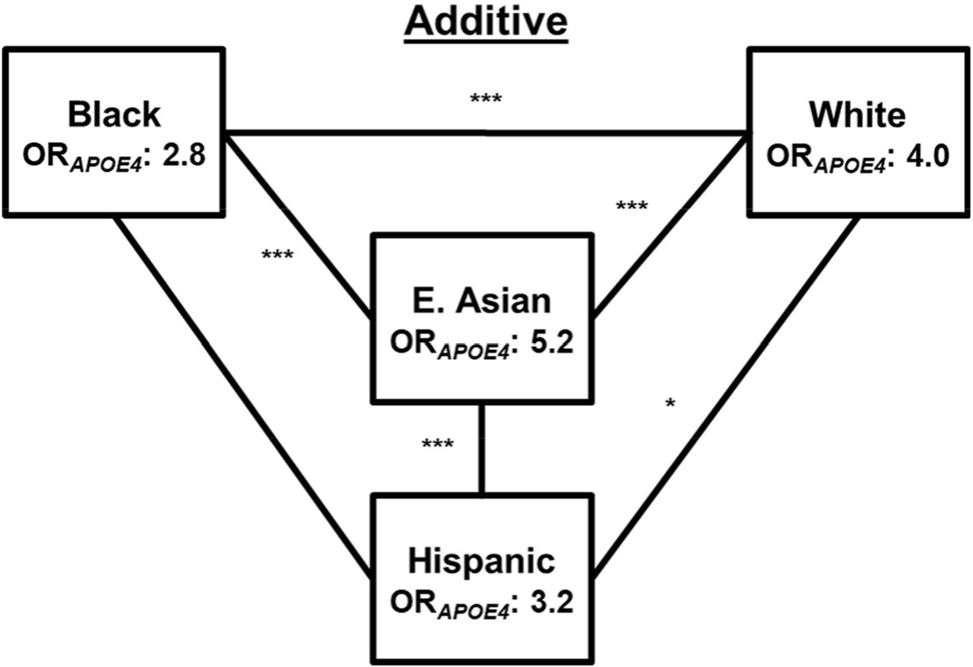
Additive OR_*APOE4*_ across multiple racial and ethnic groups. Additive *APOE4* modeling generated R/E-stratified OR_*APOE4*_. OR_*APOE4*_ generated using this model differed between all R/E groups. Statistical OR_*APOE4*_ comparisons were determined using Wald tests and are indicated by lines between the R/E groups (*: < 0.05; **: < 0.01; ***: < 0.001; no asterisk(s) indicates non-significant difference(s))

Case–control, multivariable logistic regression analyses assessed the effect of *APOE4* adjusted for age, sex, and global ancestry represented by the first two PCs. We first conceptualized *APOE4* effects using an additive model with *APOE4* non-carriers as the referent group (Supplemental Eq. 1). Additive *APOE4* effect estimates (β_*APOE4*_) were then exponentiated to obtain *APOE4* OR (OR_*APOE4*_). The largest *APOE4* effects were observed in East Asian participants (OR_*APOE4*_: 5.2, 95%CI: 4.4–6.0) followed by *APOE4* effects estimates in White (OR_*APOE4*_: 4.0, 95%CI: 3.8–4.2), Hispanic (OR_*APOE4*_: 3.2, 95%CI: 2.6–4.0), and Black (OR_*APOE4*_: 2.8, 95%CI: 2.6–3.1) participants (Table 2). All pairwise R/E OR_*APOE4*_ comparisons were evaluated and differed significantly (*P* < 0.05) except for the comparison between Black and Hispanic participants (*P* = 0.13) (Fig. 1, Table 3).

### Genotypic model

**Fig. 2:**
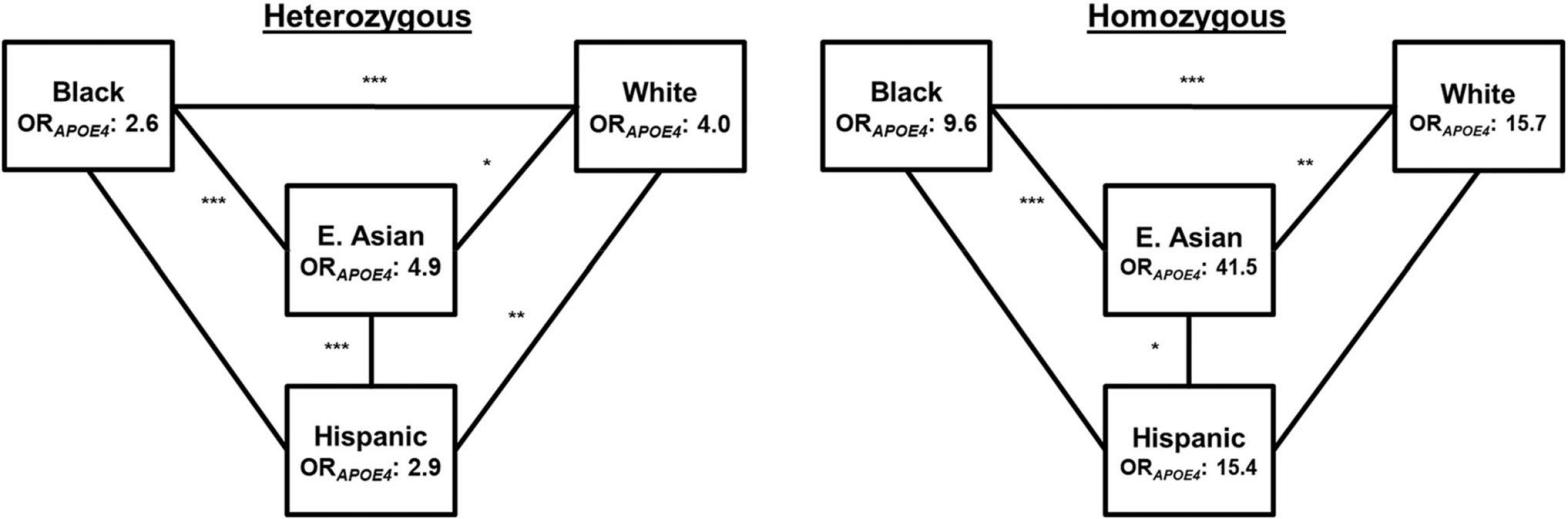
Genotypic OR_*APOE4*_ across multiple racial and ethnic groups. Heterozygote (a) and homozygote (b) OR_*APOE4*_ generated in R/E-stratified analyses. Statistical OR_*APOE4*_ comparisons were determined using Wald tests and are indicated by lines between the R/E groups for heterozygotes (a) and homozygotes (b) (*: < 0.05; **: < 0.01; ***: < 0.001; no asterisk(s) indicates non-significant difference(s)). *APOE4* non-carriers were the referent group

Next, we analyzed *APOE4* effects using a genotypic model (Supplemental Eq. 2). Participants were separated into three non-overlapping categories based on *APOE4* genotype: those with either zero, one, or two copies of the ε4 allele, as done in previous studies (Belloy, et al. 2023; Farrer, et al. 1997). Relative to *APOE4* non-carriers (*XX*), heterozygote *APOE4* ORs (*X4*) were again largest in East Asian (OR_*APOE4*_: 4.9, 95%CI: 4.1–5.8) participants followed by White (OR_*APOE4*_: 4.0, 95%CI: 3.8–4.2), Hispanic (OR_*APOE4*_: 2.9, 95%CI: 2.2–3.7), and Black (OR_*APOE4*_: 2.6, 95%CI: 2.3–2.9) participants (Table 2). All differences between R/E heterozygotes differed significantly except between Black and Hispanic participants. (Fig. 2, Table 4) Although this comparison did not reach statistical significance, Black participants trended lower OR_*APOE4*_ (*P* = 0.23).

Homozygous *APOE4* carriers (*44*) showed a similar pattern to that of heterozygotes. Among homozygotes, *APOE4*’s effect in East Asian participants (OR_*APOE4*_: 41.5, 95%CI: 22.1–88.8) was higher than in White (OR_*APOE4*_: 15.7, 95%CI: 13.8–17.8), Hispanic (OR_*APOE4*_: 15.4, 95%CI: 8.2–31.3), and Black (OR_*APOE4*_: 9.6, 95%CI: 7.5–12.3) participants (Fig. 2, Tables 2, 4, 5). In contrast to the heterozygote analyses, however, homozygote OR_*APOE4*_ differed significantly between all populations except between Black and Hispanic (*P* = 0.09) and between Hispanic and White (*P* = 0.48) participants (Fig. 2, Table 4).

**Table 2:** *APOE4* odds ratios for different genetic models

Race/ethnicity	Additive		Genotypic				DA-Adjusted	
			Heterozygote		Homozygote			
	OR_*APOE4*_	95% CI	OR_*APOE4*_	95% CI	OR_*APOE4*_	95% CI	OR_*APOE4*_	95% CI
East Asian	5.15	4.43, 6.02	4.86	4.10, 5.77	41.53	22.13, 88.79	6.44	4.70, 9.42
White	4.00	3.83, 4.17	4.02	3.82, 4.24	15.65	13.84, 17.75	3.96	3.72, 4.21
Hispanic	3.23	2.62, 4.02	2.86	2.20, 3.72	15.44	8.15, 31.29	3.93	2.85, 5.59
Black	2.83	2.57, 3.12	2.58	2.28, 2.92	9.58	7.50, 12.30	3.10	2.74, 3.51

OR_*APOE4*_ from additive, genotypic, and DA-adjusted *APOE4* regression models with 95% confidence intervals (CI). *APOE4* non-carriers were the referent group in the genotypic model. ORs were generated for additive, genotypic, and DA-adjusted regression models from Supplemental Eqs. 1, 2, or 3, respectively

**Table 3:** Comparisons between race/ethnic groups of additive OR_*APOE4*_

Race/ethnicity	East Asian	White	Hispanic	Black
East Asian				
White	< 0.001			
Hispanic	< 0.001	0.03		
Black	< 0.001	< 0.001	0.13	

Pairwise comparisons between R/E groups were conducted for the additive (b) modeling strategy using Supplemental Eq. 1

**Table 4:** Comparisons between race/ethnic groups for genotypic OR_*APOE4*_

R/E	Heterozygote				Homozygote			
	E. Asian	White	Hispanic	Black	E. Asian	White	Hispanic	Black
E. Asian								
White	0.02				0.003			
Hispanic	< 0.001	0.004			0.02	0.48		
Black	< 0.001	< 0.001	0.23		< 0.001	< 0.001	0.09	

Pairwise comparisons between R/E groups were conducted for the genotypic (c) modeling strategy using Supplemental Eq. 2. *APOE4* non-carriers (*X4*) were designated as the referent group in the genotypic model

**Table 5:** Evidence of DA among multiple racial and ethnic groups

Race/Ethnicity	Measured Homozygote OR	Predicted Homozygote OR	P-value
East Asian	41.5	26.5	0.01
White	15.7	16.0	0.64
Hispanic	15.4	10.4	0.20
Black	9.6	8.0	0.17

Measured Homozygote (*44*) and predicted homozygote OR assuming additivity (PH). *APOE4* non-carriers were the referent group in analyses

To understand how additive and genotypic OR_*APOE4*_ estimates differed, we generated homozygote (*44*) OR_*APOE4*_ predictions assuming only additive contributions. These predicted homozygote (PH) OR_*APOE4*_ estimates were the square of their respective additive OR_*APOE4*_. PH OR_*APOE4*_ were highest in East Asian (OR_*APOE4*_: 26.5) participants, followed by White (OR_*APOE4*_: 16.0), Hispanic (OR_*APOE4*_: 10.4) and Black (OR_*APOE4*_: 8.0) participants (Table 5). Additive predictions underestimated calculated homozygote genotypic OR in East Asian participants by 57%. This was the only significant difference from its genotypic counterpart across the R/E groups (Table 5). This builds upon existing literature, indicating the presence of DA through an increase in *APOE4*’s homozygote effects relative to additivity (Fortea, et al. 2024).

### Deviation from additivity

**Fig. 3:**
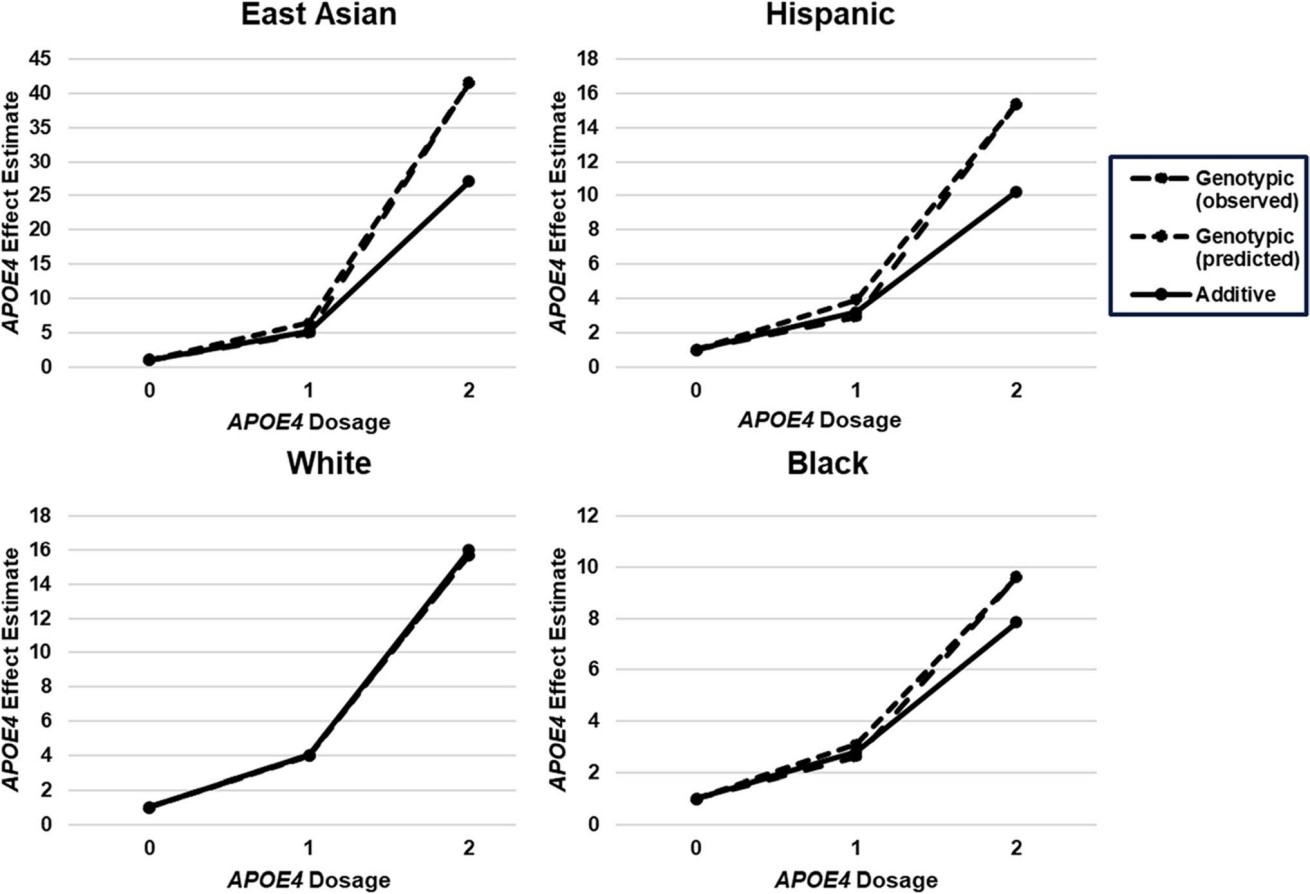
OR_*APOE4*_ differences by modeling strategy across multiple racial and ethnic groups. The impact of additive and genotypic modeling on OR_*APOE4*_. Regardless of modeling strategy, OR_*APOE4*_ estimates converge when data conforms to additive modeling assumptions. This is not observed for any non-White R/E group, indicating some degree of DA. *APOE4* non-carriers were the referent group in genotypic modeling

**Fig. 4:**
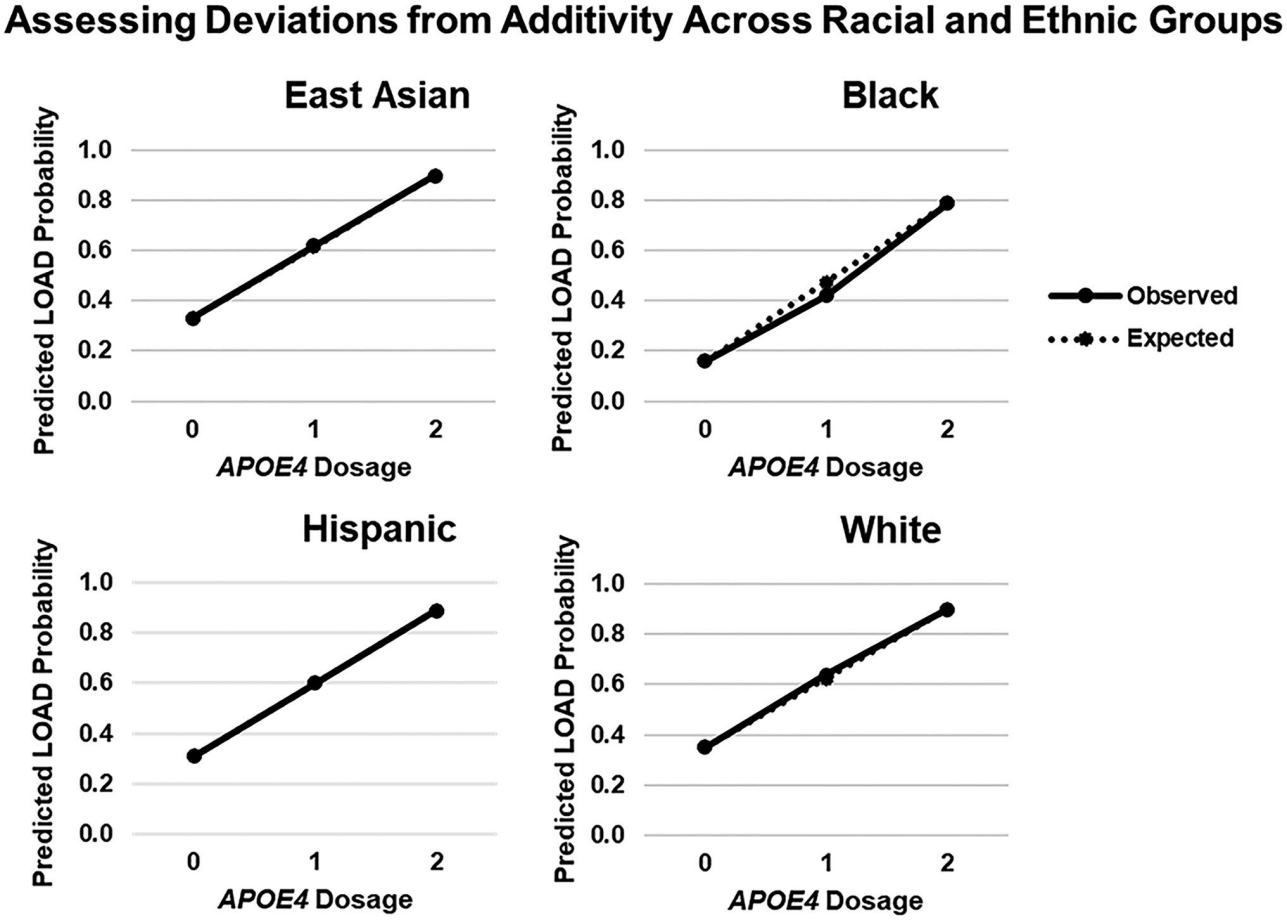
Evidence of DA across multiple racial and ethnic groups. Calculating the predicted probability of LOAD in each R/E group with increasing *APOE4* dosage in the additive model. Observed estimates deviated from predicted LOAD probabilities in Black participants, reflecting similar findings as those generated from Supplemental Eq. 3

Coupled with additional evidence of DA, we next refined the genotypic *APOE4* model by incorporating a DA adjustment into the additive *APOE4* model framework (Figs. 3 and 4, Supplemental Eq. 3). Supplemental Eq. 3 is statistically equivalent to supplemental Eq. 2, although the two approaches utilize different parametrizations of related adjustments (Supplemental Eqs. 4 and 5; Supplemental Table 2). Specifically, this approach separates *APOE4*’s effect into the *APOE4* marginal term and a DA covariate. Unlike additive or genotypic modeling, this reparameterization allows us to study the significance, magnitude, and direction of DA. Compared to additive *APOE4* ORs, Wald testing indicated non-significant changes in OR_*APOE4*_ across all R/E groups (East Asian OR_*APOE4*_: 6.4, 95%CI: 4.7–9.4; White OR_*APOE4*_: 3.9, 95%CI: 3.7–4.2; Hispanic OR_*APOE4*_: 3.9, 95%CI: 2.8–5.6; and Black OR_*APOE4*_: 3.1, 95%CI: 2.7–3.5) (Table 2). Although the differences in OR_*APOE4*_ between the additive and DA-adjusted OR_*APOE4*_ did not reach significance in any R/E group, OR_*APOE4*_ increases among East Asian, Hispanic, and Black participants approached the threshold for statistical significance (*P* = 0.13, 0.15, and 0.11, respectively). Following DA adjustment, White and Hispanic participant OR_*APOE4*_ also no longer differed from one another (Table 6). Akaike information criterion (AIC), a common model evaluation metric indicated improved model performance in East Asian, Hispanic, and Black participants with the DA term, although this improvement was significant only among Black participants (Table 7).

**Table 6:** Comparisons between race/ethnic groups for DA-adjusted OR_*APOE4*_

Race/ethnicity	East Asian	White	Hispanic	Black
East Asian				
White	** < 0.001**			
Hispanic	**0.02**	**0.48**		
Black	** < 0.001**	** < 0.001**	**0.06**	

Pairwise comparisons between R/E groups were conducted for the DA-adjusted (d) modeling strategy using Supplemental Eq. 3

**Table 7:** Model evaluation using AIC and likelihood ratio testing (LRT) for each R/E group

Race/Ethnicity	Model	AIC	LRT
East Asian	Additive	3,670.78	0.11
	DA-Adjust	3,670.27	
White	Additive	38,079.81	0.65
	DA-Adjust	38,081.61	
Hispanic	Additive	1,759.82	0.11
	DA-Adjust	1,759.21	
Black	Additive	6,684.34	0.02
	DA-Adjust	6,680.51	

Best model by AIC underlined

**Fig. 5:**
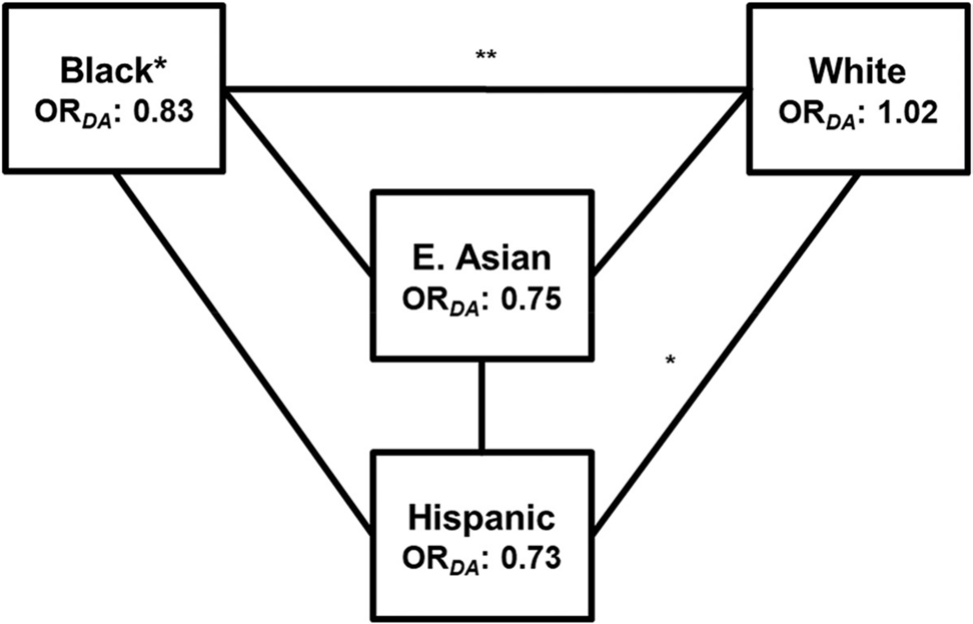
Evaluating OR_DA_ across multiple racial and ethnic groups. DA-adjusted modeling of additive *APOE4* effects generated OR_DA_ estimates for each R/E group. This estimate reached significance among Black participants. Comparisons between R/E groups indicated that these deviations only differed between Black and White participants. Statistical OR_DA_ comparisons were determined using t-tests and are indicated by lines between the R/E groups (b) (*: < 0.05; **: < 0.01; ***: < 0.001; no asterisk(s) indicates non-significant difference(s))

We then assessed the DA term in each R/E group. Reparametrizing the genotypic model while taking advantage of the additive framework provided insight into previously overlooked non-additivity in the *APOE4*-LOAD association. This approach produced population specific OR_DA_, direct and quantifiable measures of the ε4 allele, its significance, and trends toward either dominance (OR_DA_ > 1.0) or recessivity (OR_DA_ < 1.0). OR_DA_ findings converged with previous model performance results, as the DA effect again reached significance only among Black participants (OR_DA_: 0.83, 95%CI: 0.72–0.97). Estimates in the East Asian (OR_DA_: 0.75, 95%CI: 0.50–1.07) and Hispanic (OR_DA_: 0.73, 95%CI: 0.48–1.07) participants trended toward a recessive effect (Fig. 5). Although non-significant, we detected a very slight trend toward dominance among White participants (OR_DA_: 1.02, 95%CI: 0.94–1.09), the only finding across several R/E groups indicating non-recessive behavior of the ε4 allele. Finally, by comparing OR_DA_ estimates between R/E groups, we found significant differences between Black and White (*P* < 0.001) and between White and Hispanic (*P* =0.05) participants (Fig. 5, Table 8). Despite this covariate reaching significance only in the Black population, this report introduces evidence quantifying patterns of *APOE4* dosage effects initially noted by Corder et al. explicitly across several diverse populations (Corder, et al. 1993).

**Table 8:** OR_DA_ for each race/ethnicity and pairwise comparison between groups (p-values) (below diagonal)

Race/ethnicity	East Asian	White	Hispanic	Black
OR_DA_	0.75 (0.50,1.07)	1.02 (0.94,1.09)	0.73 (0.48,1.07)	0.83 (0.72,0.97)
East Asian				
White	0.95			
Hispanic	0.44	0.05		
Black	0.71	< 0.001	0.74	

The ADGC data is an amalgamation of data from 43 distinct studies, and some of these components focused their recruitment on one specific R/E, so we investigated the potential for bias due to possible impacts of collection site. We lack most information on ascertainment strategies but emphasize that our results were similar when we used mixed effect logistic regression and included a random intercept for collection site (Supplemental Table 3), indicating the results were not affected by variation among study sites.

While we were limited in terms of available covariates, we then explored if these race specific patterns might be explained by one key covariate: sex. First, we ran a race-agnostic interaction analysis in the full dataset across all RE by adding a DA-sex interaction term to Supplemental Eq. 3. We found no evidence that the DA term differed by sex (p = 0.99). Then we reran Supplemental Eq. 3 within 8 strata (males and females of the 4 R/Es). The race specific DA patterns appeared roughly similar, except that the DA terms were generally smaller in the males of each race. Among East Asians, Blacks and Hispanics males, the DA ORs differed from unity, consistent with a recessive model for *APOE4* effects. This difference was most prominent in East Asians and Hispanics. Of note, in whites where the sample size was the largest there appeared to be no deviation from additivity. Thus, the role of sex in determining deviation from additivity merits attention in future research, and other LOAD co-factors not available in this study (e.g. educational attainment, decade of birth, etc.) should also be considered.

Limitations of this observational study included the non-random population sample. As with all case–control approaches, study entry was dependent on disease status, and this creates the opportunity for unrecognized selection biases. This may introduce concerns of selection bias that are typical of such studies. The utilization of R/E potentially limits this study’s ability to accurately understand the *APOE4*-LOAD association as this socially defined population descriptor often collapses ancestrally diverse groups into a single category. While R/E stratification is one of the most common strategies for examining population differences, this approach neglects within-group heterogeneity. For example, due to the nature of R/E data collection, “Hispanic” individuals are designated as a single group despite differing genetic admixture profiles (Adhikari, et al. 2016; Granot-Hershkovitz, et al. 2021). Further, PC adjustment may not sufficiently address intra-population variation described by a single R/E identifier, e.g. Hispanic participants ascertained in the Caribbean, Mexico, and the United States. PC adjustment also fails to account for local ancestry surrounding the *APOE* gene locus that has been shown to modify LOAD risk (Rajabli, et al. 2018).

## Conclusion

In summary, this report assessed the *APOE4*-LOAD association, demonstrating findings similar, but not identical, to estimates of *APOE4*’s varying contribution to LOAD risk across multiple R/E groups compared to previous publications. The relative OR_*APOE4*_ order between R/E groups described in this report (Black < Hispanic < White < East Asian) differed from Belloy et al.’s 2023 findings (Hispanic < Black < White < East Asian) despite substantial overlap in participant data (Belloy, et al. 2023). This and other differences in findings may be due to characteristics of the non-overlapping participant samples, methods of ancestry adjustment, the impact of DA adjustment, and/or insufficient power to detect signal among East Asian, Hispanic, and, to a lesser degree Black participants compared to their White counterparts (Belloy, et al. 2023; Burton, et al. 2009; Farrer, et al. 1997; Fortea, et al. 2024; Haines 1991; Ohta, et al. 2024; So and Sham 2011; Tsouris, et al. 2024). Overall, both Belloy et al 2023 and our study suffered from relatively small samples sizes non-Europeans, but our sample sizes were even smaller for the non-Europeans. This may account for some of the differences in our observations. Our findings also indicate that additive and genotypic modeling alone may not appropriately capture R/E-specific *APOE4* effects, especially in Black populations. We detected significant DA effects in Black participants and found that DA adjustment in this R/E group produced a significantly better performing model. In addition, adjusting for DA partially ablated OR_*APOE4*_ differences in the ε4 allele’s effect across R/E groups. Stated differently, some of the magnitude in differences in R/E effect sizes are related to variation in the deviation from non-additivity across groups. Finally, this study presented evidence that DA differed between Black and White and between White and Hispanic participants, introducing evidence of population specific DA in the *APOE4*-LOAD association. Our results indicate revisiting *APOE4* GWAS estimates for all studies investigating Black populations and that future *APOE*-stratified analyses should evaluate non-additive contributions of APOE. Considering non-additivity appears to be important for most genetic analyses of LOAD, especially in Black populations. Therefore, polygenic risk scores should consider deviations from additivity, at least for APOE, the most prominent genetic risk factor for LOAD.

## Supplementary Information

Below is the link to the electronic supplementary material.

Supplementary file1 (PDF 51 KB)

Supplementary file2 (TIF 295 KB)

Supplementary file3 (TIF 2782 KB)

Supplementary file4 (DOCX 17 KB)

Supplementary file5 (DOCX 19 KB)

Supplementary file6 (DOCX 17 KB)

Supplementary file7 (DOCX 17 KB)

Supplementary file8 (DOCX 16 KB)

## Data Availability

ADGC data is available upon request from the Consortium (https://www.adgenetics.org/content/feedback-and-queries).
