# Supplementary figures and images for: Deviations from additivity in APOE4-mediated late-onset Alzheimer’s disease risk across races and ethnicities
Source: Hum Genet. 2026 Jan 22;145(1):16. doi: 10.1007/s00439-025-02810-5 (PMC12827419; doi:10.1007/s00439-025-02810-5)

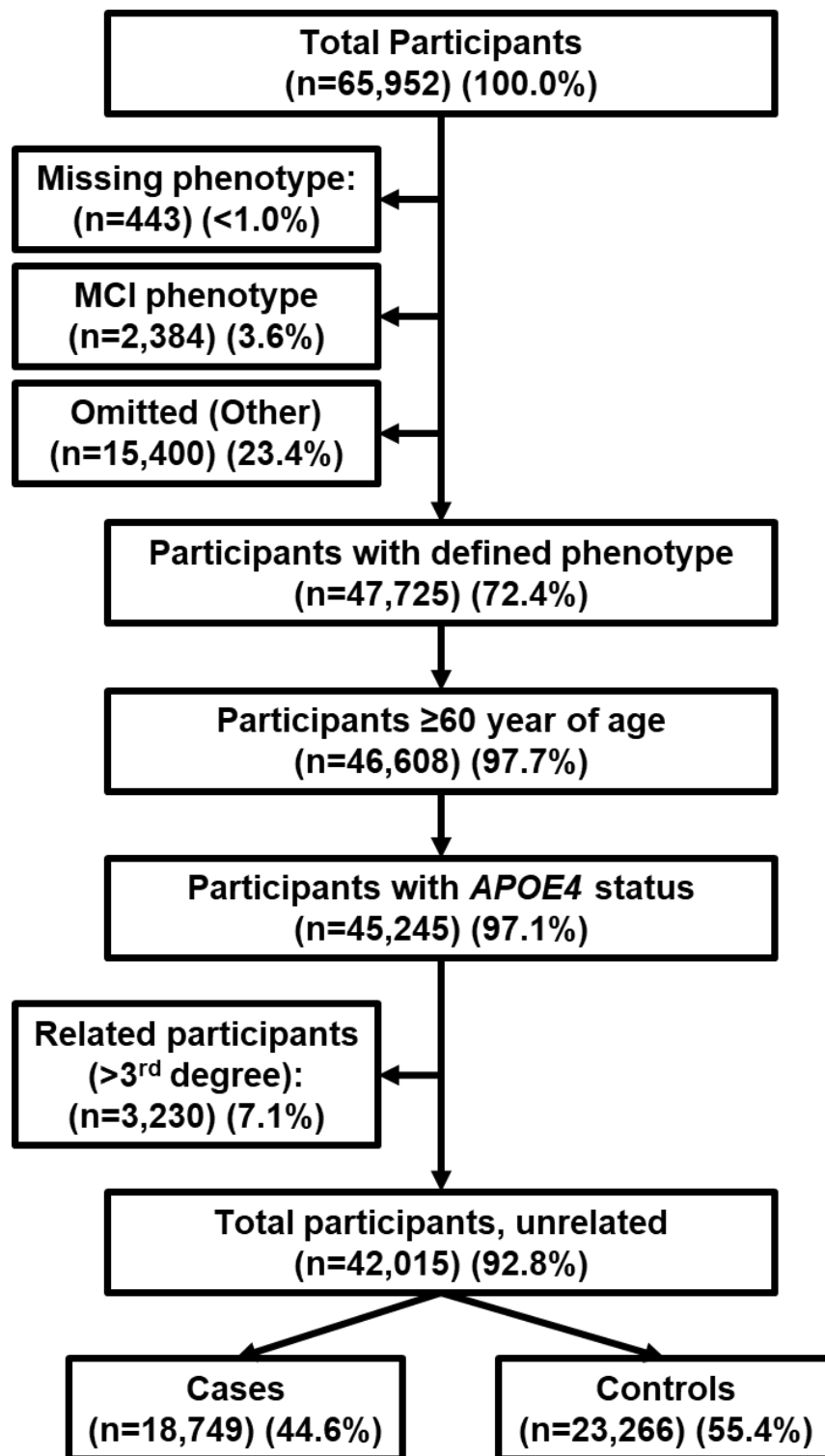

Supplement: Supplementary file 1 — Supplementary file1 (PDF 51 KB) [file 439_2025_2810_MOESM1_ESM.pdf]

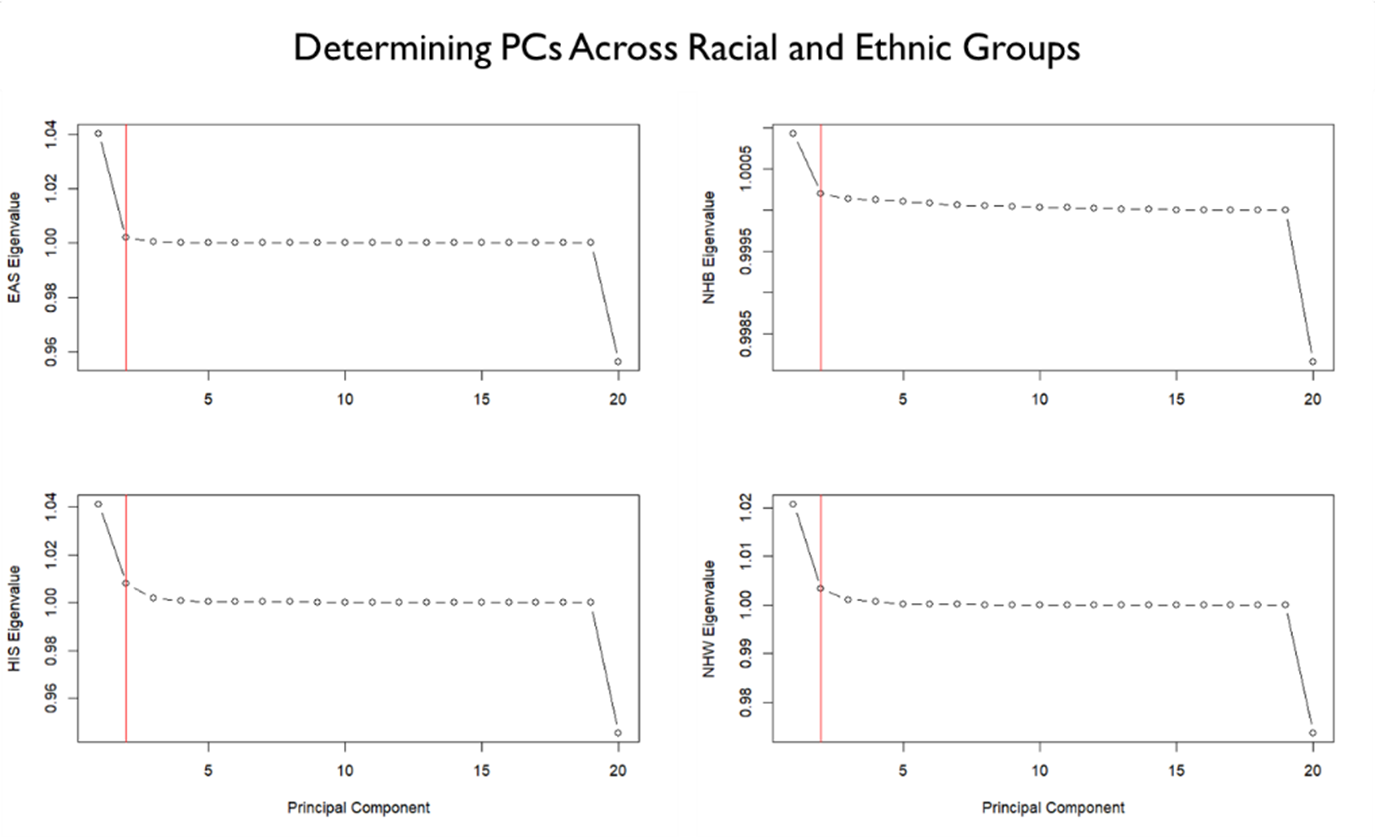

Supplement: Supplementary file 2 — Supplementary file2 (TIF 295 KB) [file 439_2025_2810_MOESM2_ESM.tif]

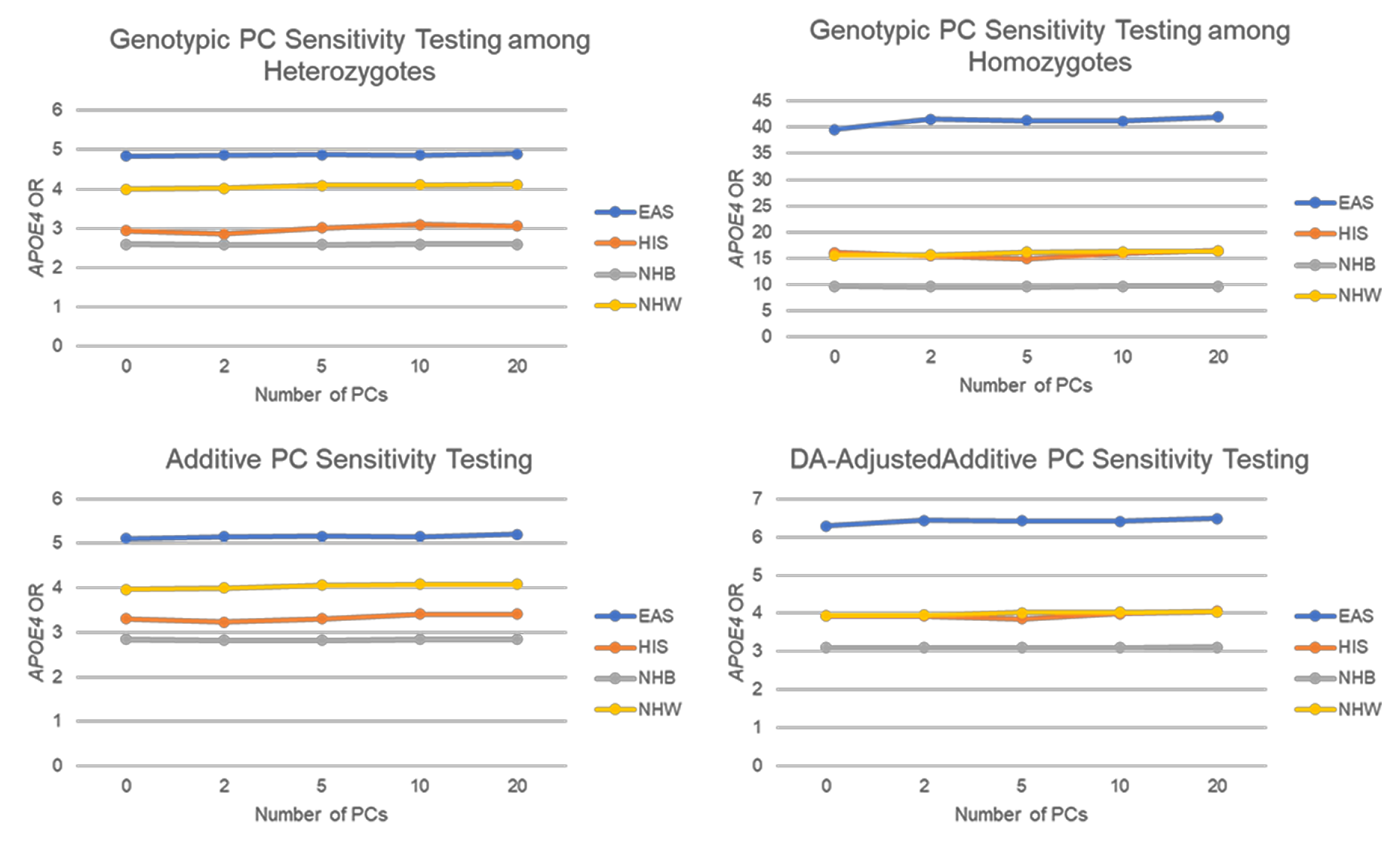

Supplement: Supplementary file 3 — Supplementary file3 (TIF 2782 KB) [file 439_2025_2810_MOESM3_ESM.tif]
