# Supplementary material for: Deviations from additivity in APOE4-mediated late-onset Alzheimer’s disease risk across races and ethnicities
Source: Hum Genet. 2026 Jan 22;145(1):16. doi: 10.1007/s00439-025-02810-5 (PMC12827419; doi:10.1007/s00439-025-02810-5)
Supplement: Supplementary file 4 — Supplementary file4 (DOCX 17 KB) [file 439_2025_2810_MOESM4_ESM.docx]

Supplemental Table 1. Cohort Sources

| Race/Ethnicity | Cohort | Number |
| --- | --- | --- |
| East Asian | ADC10 | 192 |
|  | ADC11 | 30 |
|  | ADC12 | 26 |
|  | ASA_JPN | 1210 |
|  | JPN | 1738 |
| White | ACT1 | 1971 |
|  | ACT3 | 1131 |
|  | ADC1 | 1986 |
|  | ADC2 | 784 |
|  | ADC3 | 1232 |
|  | ADC4 | 641 |
|  | ADC5 | 756 |
|  | ADC6 | 536 |
|  | ADC7 | 1293 |
|  | ADC8 | 885 |
|  | ADC9 | 1414 |
|  | ADC10 | 1023 |
|  | ADC11 | 892 |
|  | ADC12 | 628 |
|  | ADNI | 440 |
|  | BIOCARD | 116 |
|  | CHAP2 | 170 |
|  | EAS | 150 |
|  | GSK | 1362 |
|  | LOAD | 1830 |
|  | MAYO | 1657 |
|  | MIRAGE | 682 |
|  | OHSU | 274 |
|  | Rmayo | 215 |
|  | ROSMAP1 | 905 |
|  | ROSMAP2 | 122 |
|  | TARCC1 | 491 |
|  | TARCC3 | 145 |
|  | TGEN2 | 961 |
|  | UKS | 598 |
|  | UMVUMSSM | 2216 |
|  | UMVUTARC2 | 348 |
|  | UPITT | 2038 |
|  | WASHU1 | 494 |
|  | WASHU2 | 108 |
|  | WHICAP | 611 |
| Hispanic | ACT3 | 23 |
|  | ADC8 | 21 |
|  | ADC9 | 269 |
|  | ADC10 | 45 |
|  | ADC11 | 314 |
|  | ADC12 | 13 |
|  | PRADI | 638 |
|  | TARCC3 | 303 |
|  | TARCC4 | 20 |
| Black | ACT | 86 |
|  | ADC1-2 | 110 |
|  | ADC3 | 204 |
|  | ADC8 | 637 |
|  | ADC9 | 305 |
|  | ADC11 | 386 |
|  | CHAP | 512 |
|  | CHOP | 1425 |
|  | INDIANAPOLIS | 1121 |
|  | JHU | 391 |
|  | NIALOAD_NCRAD | 47 |
|  | REAAADI | 844 |
