# Supplementary material for: Deviations from additivity in APOE4-mediated late-onset Alzheimer’s disease risk across races and ethnicities
Source: Hum Genet. 2026 Jan 22;145(1):16. doi: 10.1007/s00439-025-02810-5 (PMC12827419; doi:10.1007/s00439-025-02810-5)
Supplement: Supplementary file 5 — Supplementary file5 (DOCX 19 KB) [file 439_2025_2810_MOESM5_ESM.docx]

**Supplemental Table 2. Genotypic versus DA-Adjusted Reparameterization of *APOE4***

Reparameterization of Equation 3 generated *APOE4* effect estimates the same as those produced by the genotypic model (Equation 2). *APOE4* status parameterization by modeling strategy (A), establish the relationship between genotypic and DA-adjusted parameterizations (B), and demonstrate comparable *APOE4* betas generated using genotypic or DA-adjusted regression analyses (C).

A. *APOE4* status parameterization by modeling strategy.

|  | **Genotypic** | | **DA-Adjusted** | |
| --- | --- | --- | --- | --- |
|  | **β_X4_^1^** | **β_44_** | **α_A_^2^** | **α_D_** |
| ***XX*** | **0** | **0** | **0** | **0** |
| ***X4*** | **1** | **0** | **1** | **1** |
| ***44*** | **0** | **1** | **2** | **0** |

1 - β_X4_ and β_44_ refer to heterozygote or homozygote OR_APOE4_ effect estimates generated from genotypic genetic models, respectively.

2 - α_A_ and α_D_ refer to OR_APOE4_ and OR_DA_ generated from DA-adjusted genetic models, respectively.

B. The relationship between genotypic and DA-adjusted parameterizations.

|  | **Genotypic** | **DA-Adjusted** |
| --- | --- | --- |
| ***APOE4***  **Non-Carriers** | **(referent)**  **β_X4_*[0] + β_44_*[0] = 0** | **α_A_*[0] + α_D_*[0] = 0** |
| ***APOE4* Heterozygotes** | **β_X4_*[1] + β_44_*[0] = β_X4_** | **α_A_*[1] + α_D_*[1] = α_A_ + α_D_** |
| ***APOE4* Homozygotes** | **β_X4_*[0] + β_44_*[1] = β_44_** | **α_A_*[2] + α_D_*[0] = 2α_A_** |

β_X4_ and β_44_ are heterozygote or homozygote OR_APOE4_ effect estimates generated from genotypic genetic models, respectively.

α_A_ and α_D_ refer to OR_APOE4_ and OR_DA_ generated from DA-adjusted genetic models, respectively.

C. Comparable *APOE4* betas generated using genotypic or DA-adjusted regression analyses

|  | **β_X4_ = α_A_+ α_D_** | | **β_44_ = 2α_A_** | |
| --- | --- | --- | --- | --- |
|  | **Genotypic** | **DA-Adjusted** | **Genotypic** | **DA-Adjusted** |
|  | **β_X4_** | **α_A_+ α_D_** | **β_44_** | **2α_A_** |
| **East Asian** | **1.6** | **1.9 - 0.3 = 1.6** | **3.7** | **2(1.9) = 3.8** |
| **White** | **1.4** | **1.4 - 0.0 = 1.4** | **2.8** | **2(1.4) = 2.8** |
| **Hispanic** | **1.1** | **1.4 - 0.3 = 1.1** | **2.7** | **2(1.4) = 2.8** |
| **Black** | **0.9** | **1.1 - 0.2 = 0.9** | **2.3** | **2(1.1) = 2.2** |

β*_X4_* and β*_44_* refer to heterozygote or homozygote OR*_APOE4_* effect estimates generated from genotypic genetic models, respectively.

α_A_ and α_D_ refer to OR*_APOE4_* and OR_DA_ generated from DA-adjusted genetic models, respectively.
