# Supplementary material for: Deviations from additivity in APOE4-mediated late-onset Alzheimer’s disease risk across races and ethnicities
Source: Hum Genet. 2026 Jan 22;145(1):16. doi: 10.1007/s00439-025-02810-5 (PMC12827419; doi:10.1007/s00439-025-02810-5)
Supplement: Supplementary file 6 — Supplementary file6 (DOCX 17 KB) [file 439_2025_2810_MOESM6_ESM.docx]

**Supplemental Table 3. *APOE4* and DA results after adding a random intercept for study site to Supplemental Equation 3**

| **Race/Ethnicity** | **OR*_APOE4_*** | **95% CI** | **OR_DA_** | **95% CI** |
| --- | --- | --- | --- | --- |
| East Asian | 6.49 | 4.60, 9.15 | 0.75 | 0.52, 1.08 |
| White | 3.91 | 3.66, 4.17 | 1.03 | 0.95, 1.11 |
| Hispanic | 4.06 | 2.77, 5.96 | 0.68 | 0.43, 1.06 |
| Black | 3.19 | 2.80, 3.62 | 0.79 | 0.67, 0.92 |
