# Supplementary material for: Deviations from additivity in APOE4-mediated late-onset Alzheimer’s disease risk across races and ethnicities
Source: Hum Genet. 2026 Jan 22;145(1):16. doi: 10.1007/s00439-025-02810-5 (PMC12827419; doi:10.1007/s00439-025-02810-5)
Supplement: Supplementary file 7 — Supplementary file7 (DOCX 17 KB) [file 439_2025_2810_MOESM7_ESM.docx]

**Supplemental Table 4. *APOE4* and DA results from Supplemental Equation 3 after stratifying by sex and R/E**

|  | **Females** | | **Males** | |
| --- | --- | --- | --- | --- |
| **Race/Ethnicity** | **OR_DA_** | **95% CI** | **OR_DA_** | **95% CI** |
| East Asian | 1.05 | 0.66, 1.57 | 0.44 | 0.17, 0.83 |
| White | 1.05 | 0.95, 1.15 | 0.98 | 0.88, 1.10 |
| Hispanic | 0.84 | 0.53, 1.31 | 0.35 | 0.08, 0.87 |
| Black | 0.88 | 0.74, 1.05 | 0.74 | 0.56, 0.97 |
