# Supplementary material for: Deviations from additivity in APOE4-mediated late-onset Alzheimer’s disease risk across races and ethnicities
Source: Hum Genet. 2026 Jan 22;145(1):16. doi: 10.1007/s00439-025-02810-5 (PMC12827419; doi:10.1007/s00439-025-02810-5)
Supplement: Supplementary file 8 — Supplementary file8 (DOCX 16 KB) [file 439_2025_2810_MOESM8_ESM.docx]

Equation 1.

$$logit\left( Probaility of LOAD \right)= \alpha+\beta_{APOE4}*g_{1}+ \beta_{Age}*g_{2}+\beta_{Sex}*g_{3} + \beta_{PC1}*g_{4} + \beta_{PC2}*g_{5}$$

α: intercept; β*_APOE4_*: additive *APOE4* effect estimate; g_1_: *APOE4* status (continuous); β*_Age_*: age effect estimate; g_2_: age at onset (cases), age at last examination (controls) (continuous); β*_Sex_*: sex effect estimate; g_3_: sex (female / male) (binary); β*_PC1_*: effect estimate of the first PC; g_4_: first PC (continuous); β*_PC2_*: effect estimate of the second PC; g_5_: second PC (continuous). Betas produced by this regression are exponentiated to obtain covariate ORs.

Equation 2.

$$logit\left( Probaility of LOAD \right)= \alpha+\beta_{APOE4}*g_{1}+ \beta_{Age}*g_{2}+\beta_{Sex}*g_{3} + \beta_{PC1}*g_{4} + \beta_{PC2}*g_{5}$$

α: intercept; ; β*_APOE4_*: genotypic *APOE4* effect estimate; g_1_: *APOE4* status (multi-level categorical; referent group: *APOE4* non-carriers (*XX)*); β*_Age_*: age effect estimate; g_2_: age at onset (cases), age at last examination (controls) (continuous); β*_Sex_*: sex effect estimate; g_3_: sex (female/male) (binary); β*_PC1_*: effect estimate of the first PC; g_4_: first PC (continuous); β*_PC2_*: effect estimate of the second PC; g_5_: second PC (continuous). Betas produced by this regression are exponentiated to obtain covariate ORs.

Equation 3.

$$logit\left( Probaility of LOAD \right)= \alpha+\beta_{\mathrm{DA}}*g_{1}+\beta_{APOE4}*g_{2}+ \beta_{Age}*g_{3}+\beta_{Sex}*g_{4} + \beta_{PC1}*g_{5} + \beta_{PC2}*g_{6}$$

α: intercept; β*_DA_*: DA effect estimate; g_1_: DA adjustment (binary; *APOE4* non-carriers (*XX*) and *APOE4* homozygotes (*44*) (combined group) / *APOE4* heterozygotes (*X4*)); β*_APOE4_*: additive *APOE4* effect estimate; g_2_: *APOE4* status (continuous); β*_Age_*: age effect estimate; g_3_: age at onset (cases), age at last examination (controls) (continuous); β*_Sex_*: sex effect estimate; g_4_: sex (female / male) (binary); β*_PC1_*: effect estimate of the first PC; g_5_: first PC (continuous); β*_PC2_*: effect estimate of the second PC; g_6_: second PC (continuous). Betas produced by this regression are exponentiated to obtain covariate ORs. This model is equivalent to Equation 2 but uses a different parametrization.

Equation 4.

$$logit(Probability of LOAD)= {\alpha+\beta}_{X4}g_{X4}+\beta_{44}g_{44}$$

Simplified regression model assessing the genotypic parameterization of *APOE4*. β*_X4_*: heterozygote *APOE4* (*X4*) effect estimate; g*_X4_*: heterozygote *APOE4* status (binary; *APOE4* non-carriers (*XX*) and *APOE4* homozygotes (*44*) (combined group) (referent) / *APOE4* heterozygotes (*X4*)); β*_44_*: homozygote *APOE4* (*44*) effect estimate; g*_44_*: homozygote *APOE4* status (binary; *APOE4* non-carriers (*XX*) and *APOE4* heterozygotes (*X4*) (combined group) (referent) / *APOE4* homozygotes (*44*)).

Equation 5.

$$logit(Probability of LOAD)= {\alpha+\beta}_{A}g_{A}+{\alpha\beta}_{D}g_{D}$$

Simplified regression model assessing the DA-adjusted parameterization of *APOE4*. β_A_: additive *APOE4* effect estimate; g*_A_*: additive *APOE4* status (continuous); β_D_: DA effect estimate; g*_D_*: DA adjustment (binary; *APOE4* non-carriers (*XX*) and *APOE4* homozygotes (*44*) (combined group) (referent) / *APOE4* heterozygotes (*X4*)).
